# Spanish Women Making Risky Decisions in the Social Domain: The Mediating Role of Femininity and Fear of Negative Evaluation

**DOI:** 10.3389/fpsyg.2020.561715

**Published:** 2020-12-18

**Authors:** Laura Villanueva-Moya, Francisca Expósito

**Affiliations:** Department of Social Psychology, Mind, Brain and Behavioral Research Center (CIMCYC), Faculty of Psychology, University of Granada, Granada, Spain

**Keywords:** risk-taking, femininity, fear of negative evaluation, gender roles, sex differences, gender stereotypes

## Abstract

Authors have empirically evidenced that cultural stereotypes influence gender-typed behavior. With the present work, we have added to this literature by demonstrating that gender roles can explain sex differences in risk-taking, a stereotypically masculine domain. Our aim was to replicate previous findings and to analyze what variables affect women making risky decisions in the social domain. A sample composed of 417 Spanish participants (281 women and 136 men), between 17 and 30 years old (*M* = 22.34, *SD* = 3.01), answered a set of self-report measures referring to femininity, fear of negative evaluation, and social risk-taking. According to the main results, sex indirectly linked to risk-taking in the social domain, through femininity and fear of negative evaluation. Specifically, women (vs. men) self-reported higher feminine traits, which were associated with increased fear of negative evaluation, which in turn was associated with less risky decisions in the social domain. Thus, we have showed the relationship between gender roles and women’s behaviors in a stereotypically masculine domain (risk-taking). Our findings highlight the necessity of considering a gender-based perspective in the field of risk-taking, showing that not all women make more risky decisions in the social domain.

## Introduction

Notwithstanding an increase in women and men occupying nontraditional domains, gender stereotypes are still present in society and influence women’s and men’s behaviors (Eagly and Wood, 2016). Gender stereotypes have sustained gender inequality (Ellemers, 2018), limiting women to stereotypically feminine activities (i.e., the private sphere; Eagly and Wood, 2016) and discriminating against them if they do not carry out these types of activities (Rudman et al., 2012). In this respect, empirical evidence has shown sex differences in decision-making (a stereotypically masculine domain; Morgenroth et al., 2018), namely that women make fewer risky decisions than men (e.g., Figner and Weber, 2011; Van den Bos et al., 2013). Nevertheless, when some real-life domains of risk-taking are taken into account, researchers have demonstrated that men make more risky decisions in all domains except the social domain, where women make more risky decisions (e.g., Blais and Weber, 2006; Lozano et al., 2017; Morgenroth et al., 2018). Recent studies have tried to explain these differences, demonstrating that they should be interpreted with caution. For example, Rolison and Shenton (2020) indicated that these differences could be due to item bias. In this study, from a gender-based perspective, we proposed that these differences could be due to the influence of other variables, such as femininity and fear of negative evaluation. In this sense, previous research has shown traditional gender roles (femininity) increase the preference for stereotypically feminine domains (e.g., Dinella et al., 2014). Furthermore, femininity seems to restrict social behavior; Cella et al. (2013) found that femininity increased social insecurity. Hence, persons who identify themselves as more feminine – usually women – seem to be concerned about others’ expectations of them, given that they have to behave in a manner consistent with their gender role (Eagly, 1987). Consequently, their behavior seems to tend to avoid the prospect of being evaluated negatively, decreasing their participation in stereotypically masculine domains, such as sports (e.g., Yi-Hsiu and Chen-Yueh, 2013). In this respect, the sensation people experience at the prospect of being evaluated negatively by others has been specified as fear of negative evaluation (FNE; Leary, 1983). In this study, we proposed broadening the research on women, gender stereotypes, and FNE in another stereotypically masculine domain: risk-taking. We have reported a gender-based perspective on how, through femininity and fear of negative evaluation, women make decisions in the stereotypically masculine domain of risk-taking.

### The Importance of Gender Stereotypes to Women

By social role theory (Eagly and Steffen, 1984; Eagly, 1987; Eagly et al., 2000), people learn that they have to behave consistently with their gender role, given that women and men are socialized into different values starting from their childhood. A prescription exists for what women and men are expected to do: Women have to behave in accordance with a communal dimension – maintenance of relationships – and men in accordance with an agency one – goal achievement and task-functioning. Despite an increase in women and men in nontraditional domains in recent years, traditional beliefs and lifestyles have not changed. Haines et al. (2016) compared the 1980s to the 2nd decade of the 21st century and did not find a decline in the traditional gender beliefs about women and men in several domains (traits, physical characteristics, occupations, gender roles, etc.). Those who hold such traditional beliefs continue to associate women with being primary caregivers and men with being primary family providers (Eagly and Steffen, 1984; Eagly and Wood, 2016). In this way, gender roles maintain the hegemony of patriarchy and justify the subordination of women (Ellemers, 2018), obstructing their personal and professional development (Craig and Mullan, 2011; Llinares-Insa et al., 2018). Hence, women are the main group affected by this patriarchal system in which gender roles limit their behavior and therefore interfere with their full progress and well-being.

Literature has respectively equated communal and agency dimensions with femininity (i.e., friendliness, concern for others, and expressiveness) and masculinity (i.e., mastery, independence, and competence; Bem, 1974; Abele and Wojciszke, 2014) – both gender stereotype traits. Men and women thus integrate masculinity or femininity self-concepts into themselves and self-regulate their behaviors according to them. In this regard, empirical evidence has demonstrated that women score significantly higher on self-report scales of feminine traits than men, and men higher on masculine traits than women (Kamas and Preston, 2012; López-Zafra et al., 2012; Mueller and Dato-On, 2013). Accordingly and in line with social role theory (Eagly, 1987), sex predicts feminine and masculine gender roles (e.g., Powell and Greenhaus, 2010; Ward and King, 2018; Howard and Fox, 2020); that is, persons who identify themselves as more feminine – usually women – may be expected to engage in activities related to housework, childcare, or social relationships. By contrast, persons who identify themselves as more masculine – usually men – may be expected to perform behaviors related to physically demanding or decision-making tasks (e.g., Cerrato and Cifre, 2018). Given this difference, women were our research object, and owing to gender roles affecting their personal and professional development, we used the variable of femininity as a trait that reflects women’s gender roles and so could help explain how gender roles affect their behavior in stereotypically masculine domains.

A large body of research has shown that femininity entails what women self-perceive as less competence, perpetuating gender roles in the private and public spheres (i.e., stereotypically feminine domains). Specifically, femininity predicts a family role (Powell and Greenhaus, 2010), increased interest in feminine careers or traditionally feminine jobs (Weisgram et al., 2011; Dinella et al., 2014), and decreased entrepreneurial self-efficacy (Mueller and Dato-On, 2013). At the same time, femininity affects well-being by increasing body dissatisfaction, body image concern, and depersonalization (Cella et al., 2013) as well as levels of spillover (Powell and Greenhaus, 2010). Indeed, it can affect types of strategies for managing social conflicts and increase sensibility to the needs of others, rather than decisiveness or selfishness (Keener and Strough, 2017). In line with the prior literature, we considered femininity as a possible predictor of sex differences in stereotypically masculine domains (e.g., risk-taking).

### Femininity and Fear of Negative Evaluation

Eagly and Wood (2016) argued that one of the main reasons people continue to conform to their gender roles is the negative social evaluation they could receive if they were to disregard them. Indeed, if women violate gender roles, they are perceived more negatively than a stereotypical male or female (e.g., Sutherland et al., 2015). Consequently, they fall victim to social and economic penalties (what is known as *backlash*; e.g., Rudman et al., 2012), such as prejudice and discrimination (e.g., Glick and Fiske, 1997; Rudman and Phelan, 2008), and even they can be perceived as lesbian regardless of sexual orientation (e.g., Salvati et al., 2019). In this sense, we propose that women who self-report greater feminine traits could experience more FNE, for if they were to deviate from their femininity, they could experience negative sanctions. Specifically, it has been found that people with higher FNE tend to behave in a manner to avoid the prospect of being evaluated negatively, to be more concerned about making good impressions, and to seek social approval (Watson and Friend, 1969; Leary, 1983). This sensation (FNE) could be experienced by feminine women to a greater extent and could, therefore, be a variable limiting their behavior. In this sense, Cella et al. (2013) showed that femininity restricts social behavior, increasing avoidance or social insecurity.

Most studies on women, gender stereotypes, and FNE have been in the stereotypically masculine domain of sports (e.g., Yi-Hsiu and Chen-Yueh, 2013; for a review, see Chalabaev et al., 2013). It has generally been found that women experience higher FNE than men (e.g., Piqueras et al., 2012; Biolcati, 2017), decreasing or avoiding participation in masculine sports (e.g., Yi-Hsiu and Chen-Yueh, 2013). These findings could owe to women’s concerns about not achieving social standards of femininity (Leary, 1992), given that if they were involved in stereotypically masculine domains (i.e., sports, work, decision-making…), their participation could be perceived as a deficiency in femininity, and they could receive negative sanctions. Similarly, in other stereotypically masculine domains, such as negotiations, Amanatullah and Morris (2010) demonstrated that fear of social costs affects women’s strategic responses, representing a form of backlash.

### Femininity, Fear of Negative Evaluation, and Risk-Taking

With this frame of reference, we propose broadening the research on women, gender stereotypes, and FNE in another stereotypically masculine domain: decision-making. Due to gender roles, women continue to take primary responsibility for family and childcare tasks, whereas men assume decision-making tasks (Cerrato and Cifre, 2018). In fact, empirical evidence has shown sex differences in decision-making, namely that women make fewer risky decisions than men do (e.g., Figner and Weber, 2011; for a review, see Van den Bos et al., 2013). Researchers have explained these differences by anxiety (e.g., Panno et al., 2018), stress (e.g., Santos-Ruiz et al., 2012), and even the type of information processing (e.g., Byrne and Worthy, 2016).

Specifically, the literature has also found sex differences in some real-life domains of risk-taking. These differences have appeared on the Domain-Specific Risk-Taking Scale (DOSPERT; Blais and Weber, 2006), a measure and one of the most effective clinical instruments for assessing the tendency to make risky decisions across real-life domains (ethical, health, recreational, social, and financial; Harrison et al., 2005). Researchers have demonstrated that sex predicts risk-taking (e.g., Gowen et al., 2019). Specifically, men make more risky decisions in all domains except the social domain in which women make more risky decisions (e.g., Blais and Weber, 2006; Lozano et al., 2017; Morgenroth et al., 2018). Recent studies have tried to explain these sex differences on the DOSPERT scale, demonstrating that they must be interpreted with caution. On one hand, Rolison and Shenton (2020) through two studies argued that these differences could owe to the way the domains are represented. In their first study, they asked participants to report some activities in each of the domains – that is, participants had to think about and write activities, instead of answering to the original items. In their second study, they asked participants to indicate the likelihood that they would engage in each of the activities that other participants described in the first study. Their findings indicated that in the social domain, women perceived greater risk than men; in other words, they had a lower tolerance for risk. On the other hand, Zhang et al. (2019) pointed out that risk-taking in the social domain functions differently across groups. Furthermore, other authors have argued that there is a gender confirmation bias in risk-taking due to its traditional association with stereotypically masculine activity (Morgenroth et al., 2018), which could affect women’s behavior. Therefore, sex differences in the social domain (DOSPERT) should be exhaustively analyzed, given that there is controversy around this finding. Further, not all women could make more risky decisions in the social domain.

### The Current Research

The present study aims to replicate previous findings and broaden the research on women, gender stereotypes, and risk-taking. The literature has indicated that women rate themselves more likely to make risky decisions in the social domain (e.g., Figner and Weber, 2011). Nevertheless, there is controversy around this finding (Zhang et al., 2019), which may cause confusion because people who identify themselves as more feminine – traditionally women – are conditioned to be more cautious, whereas those who identify themselves as more masculine – traditionally men – are conditioned to be riskier (Carver et al., 2013). In this sense, the social domain (e.g., “speaking your mind about an unpopular issue in a meeting at work” or “moving to a city far away from your extended family”) is a context in which women could experience more FNE if they were to make risky decisions, given that they would deviate from their traditional role (Rudman et al., 2012). Moreover, researchers have demonstrated that women make decisions taking into account all information in an environment (e.g., social sanctions), even when this information could lead them to make bad decisions (e.g., Byrne and Worthy, 2016; Meyers-Levy, 1989). Hence, women who report greater feminine traits should experience higher FNE and thus make fewer risky decisions, because if they were to be involved in stereotypically masculine domains, they could be perceived as having a deficiency in femininity and could receive negative sanctions.

On the basis of prior studies’ findings, we proposed that this gender confirmation bias in risk-taking (Morgenroth et al., 2018) could be explained through gender roles (femininity) and FNE. In this research, we replicated previous findings as well as tried to increase the knowledge on the implications of femininity for FNE in risk-taking in the social domain. The general purpose of this work is to analyze how women make risky decisions in the social domain through femininity and FNE. Specifically, we predicted that women in comparison to men would self-report greater feminine traits (Hypothesis 1a), would experience higher FNE (Hypothesis 1b), and would take greater risks in the social domain (Hypothesis 1c). Concerning correlation between variables, we hypothesized that femininity in women would be associated positively with FNE (vs. men; Hypothesis 2a) and negatively with risk-taking in the social domain (vs. men; Hypothesis 2b). We also expected that FNE would be negatively associated with risk-taking in the social domain in women (vs. men; Hypothesis 3). Finally, through a serial mediation model, we predicted that women (vs. men) would be associated with more femininity, which we expected to be associated with more FNE, which would in turn be associated with less risk-taking in the social domain (Hypothesis 4).

## Materials and Methods

### Participants

We collected data from 502 students at the University of Granada in southern Spain. The inclusion criterion was being a student of the University of Granada. Among the participants who accessed the survey, 85 were excluded (14 did not complete it and 71 failed to pass an attention check item), leaving data from 417 participants (281 females and 136 males). Participants ranged in age from 17 to 30 (*M* = 22.34, *SD* = 3.01). *A priori* power analysis of G*Power (Faul et al., 2007, 2009) revealed that we had to recruit at least 120 participants to conduct a correlation statistical test with a medium effect size of *d* = 0.25 (1 – *β* = 80%; *α* = 0.05).

### Procedure

We invited participants to take part in the study through the university mailing list for students. In the email, participants received a questionnaire link and instructions to take part by an online platform. We obtained informed consent from participants before they began the study, telling them about the anonymity and confidentiality of their responses and allowing them to agree or decline to answer the survey (“After being informed of the above, I agree to participate in the study.”). If participants agreed, they could begin to answer the measures. Informed consent was obtained from all individual participants included in the study. The study is part of a broad project approved by the Ethics Committee of the University of Granada.

### Measures

#### Femininity

For femininity, we used the Bem Sex Role Inventory (Bem, 1974), adapted to the Spanish population by López-Sáez and Morales (1995, see also López-Sáez et al., 2008). The inventory assesses the extent to which people have incorporated feminine or masculine traits into their self-concepts. In particular, we administered the femininity subscale (e.g., “Sensitive to needs of others,” “childlike,” and “compassionate”). Participants were asked to rate the extent to which items described them (1 = *never or almost never true*, 7 = *almost always true*). In the present study, the internal consistency was 0.73, similar to administrations of the measure in other Spanish samples (*α* = 0.72–0.76, López-Sáez et al., 2008; López-Zafra et al., 2012).

#### Fear of Negative Evaluation

For FNE, we used the Brief Fear of Negative Evaluation Scale (Leary, 1983; Spanish adaptation of Gallego et al., 2007), which consists of 12 items that identify the sensation people experience at the prospect of being evaluated negatively by others. Examples of items include “I am afraid that others will not approve of me” and “I often worry that I will say or do the wrong thing” (1 = *not at all characteristic of me*, 5 = *extremely characteristic of me*). The Spanish adaptation showed a Cronbach’*α* of 0.90. In this data set, averages scores showed an internal consistency of 0.87, similar to other Spanish samples (*α* = 0.91, Piqueras et al., 2012).

#### Social Risk-Taking

We used the DOSPERT scale (Blais and Weber, 2006) to evaluate the likelihood of people making risky decisions within different domains of life (ethical, financial, health, recreational, and social). Lozano et al. (2017) adapted the scale to the Spanish population. We specifically administered the social subscale, which comprises six items (e.g., “Moving to a city far away from your extended family”; 1 = *extremely unlikely*, 7 = *extremely likely*). In the original version of the scale, the Cronbach’s α coefficient ranged between 0.57 and 0.79. The Spanish adaptation of the DOSPERT obtained an internal consistency of 0.64 (Lozano et al., 2017). With this sample, the subscale showed a Cronbach’s *α* of 0.65.

#### Attention Check

We included several extra attention check items among the scales to identify subjects not paying attention to the task (e.g., “If you are reading this question, answer with 3”; Lozano et al., 2017).

### Statistical Analysis Strategy

**Figure 1:**
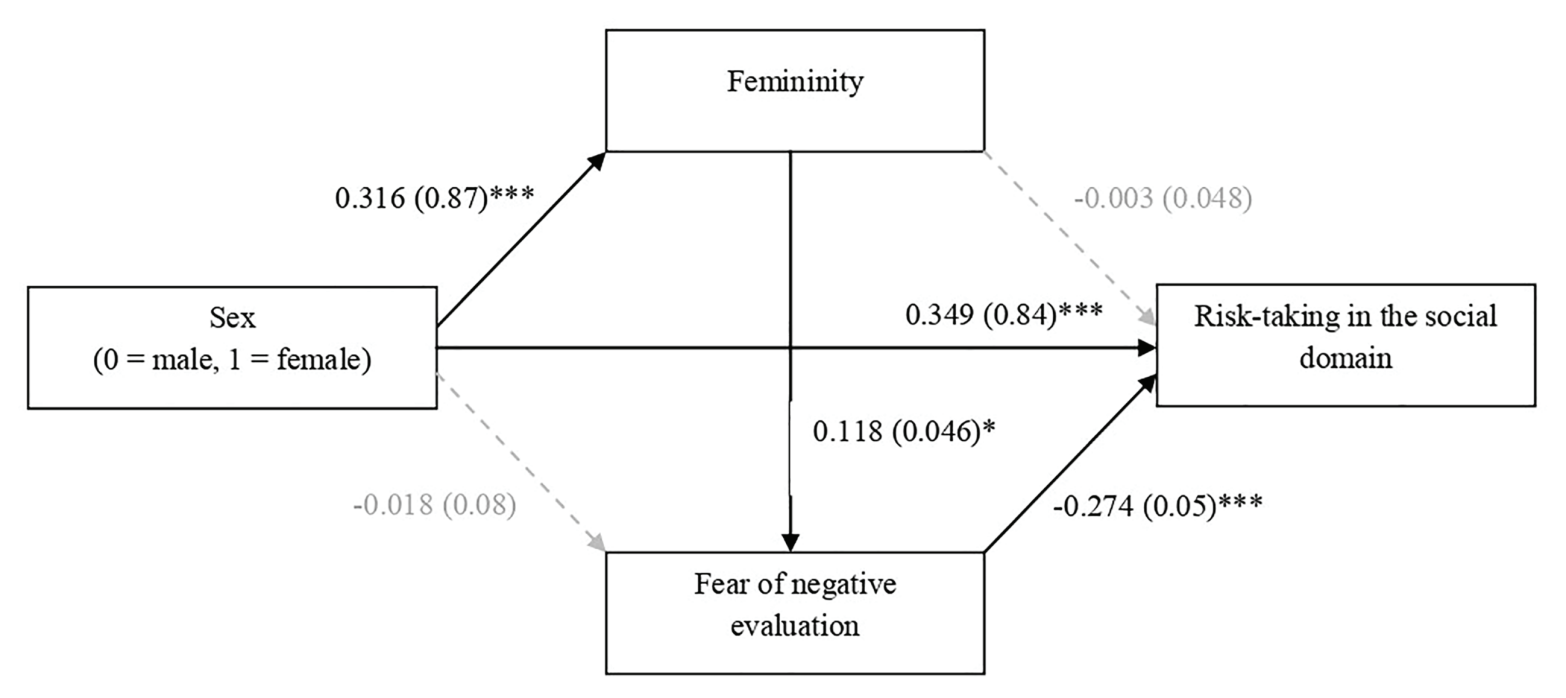
Serial mediation model depicting indirect effect sex (0 = male, 1 = female) on social risk-taking through femininity and fear of negative evaluation. Unstandardized beta coefficients reported, with standard errors within parentheses. ^*^p < 0.05, ^***^p < 0.001.

Before performing the main analysis, we checked data for testing assumptions of normality and multicollinearity. We then carried out the main analyses. To corroborate if the means of women and men were significantly different from each other in the study variables, we performed an independent samples *t*-test analysis using sex as the independent variable, and femininity, FNE, and social risk-taking as dependent variables (see Table 1). Additionally, to determine the association between the study variables, we carried out a bivariate correlation analysis as a function of sex (see Table 2). Lastly, we followed Hayes’s recommendations (2017) for testing indirect effects with serial mediators. In particular, we conducted analysis to determine whether femininity and FNE mediated the relationship between sex and social risk-taking (see Figure 1; Table 3). In particular, we used model 6 of the PROCESS macro for SPSS version 3.4.1. We performed all analyses using version 22.0 of IBM SPSS Statistics for Windows.

**Table 1:** Sex differences in femininity, fear of negative evaluation, and social risk-taking.

	Men *M* (*SD*)	Women *M* (*SD*)	*t*	*p*	95% CI	*Cohen’s d*
1. Femininity	4.57 (0.82)	4.88 (0.83)	−3.65	<0.001	[−0.486, −0.146]	0.38
2. FNE	2.99 (0.78)	3.02 (0.79)	−0.24	0.815	[−0.180, 0.142]	0.03
3. Social risk-taking	5.29 (0.88)	5.63 (0.79)	−3.99	<0.001	[−0.511, −0.174]	0.41

FNE, fear of negative evaluation; *M*, mean; *SD*, standard deviation; CI, confidence interval.

**Table 2:** Correlations and descriptive statistics across all measures.

Variables	1.	2.	3.
Femininity	-	0.20^**^	−0.13^*^
Fear of negative evaluation	−0.03	-	−0.32^**^
Social risk-taking	0.14	−0.15	-
Range	(1–7)	(1–5)	(1–7)
Observed range	(2–6.56)	(1.08–5)	(2.67–7)
Mean (*SD*)	4.78 (0.84)	3.01 (0.78)	5.52 (0.84)
**Skewness/Kurtosis**			
Women	−0.324/0.011	0.111/−0.767	−0.499/−0.287
Men	−0.194/−0.345	0.166/−0.581	−0.379/0.413

Correlations for women are above the diagonal. Correlations for men are below the diagonal. *SD*, standard deviation.

* *p* < 0.05;

** *p* < 0.01.

**Table 3:** Serial mediation analysis of sex, femininity, and fear of negative evaluation on social risk-taking.

Antecedent	Femininity		FNE^b^		Social risk-taking	
	Coeff.	Symmetric BCI^c^	Coeff.	Symmetric BCI^c^	Coeff.	Symmetric BCI^c^
Sex^a^	0.316^***^	[0.146, 0.486]	−0.018	[−0.181, 0.145]	0.349^***^	[0.183, 0.515]
Femininity			0.118^*^	[0.028, 0.209]	−0.003	[−0.096, 0.090]
FNE^b^					−0.274^***^	[−0.373, −0.176]
*Constant*	4.566^***^	[4.427, 4.706]	2.458^***^	[2.023, 2.893]	6.122^***^	[5.617, 6.628]
	*R*^2^ = 0.031		*R*^2^ = 0.016		*R*^2^ = 0.103	
	*F*(1, 415) = 13.32, *p* < 0.001		*F*(2, 414) = 3.31, *p* = 0.038		*F*(3, 413) = 15.81, *p* < 0.001	

a 0, male; 1, female.

b FNE, fear of negative evaluation.

c Symmetric BCI, symmetric bootstrapping confidence interval.

* *p* < 0.05;

*** *p* < 0.001.

## Results

### Preliminary Analysis

Skewness and kurtosis values were reported in Table 2. According to Blanca et al. (2013) the values were <1.0 and thus the assumption of normality was fulfilled. As can be observed in Table 2, correlations ranged from *r* = |0.03| to *r* = |0.32|, and thus they were not >0.70–0.80, indicating that there was no multicollinearity (Slinker and Glantz, 1985).

### Sex Differences

We conducted an independent samples *t*-test analysis to test whether women compared to men would self-report greater feminine traits (Hypothesis 1a), experience higher FNE (Hypothesis 1b), or score higher on risk-taking in the social domain (Hypothesis 1c). We used sex (0 = male; 1 = female) as the independent variable and femininity, FNE, and social risk-taking as dependent variables. As can be observed in Table 1, women self-reported greater feminine traits (Hypothesis 1a) and social risk-taking than men did (Hypothesis 1c). Conversely, with respect to FNE, the results did not show statistically significant differences based on participants’ sex and thus did not support Hypothesis 1b.

### Correlations Across All Measures

To check associations between study variables, we performed a bivariate correlation analysis as a function of sex. In Table 2, correlations for women are shown above the diagonal, whereas those for men are shown below the diagonal. The results revealed that in women (vs. men), femininity was related positively to FNE (*r* = 0.20, *p* < 0.01; Hypothesis 2a) and negatively to social risk-taking (*r* = −0.13, *p* < 0.05; Hypothesis 2b). Further, FNE in women was negatively associated with social risk-taking (*r* = −0.32, *p* < 0.01; Hypothesis 3). In men, there were no significant correlations between variables. We used Fisher’s r-to-z transformation for independent samples to determine whether there was a significant difference between correlation coefficients (Eid et al., 2011). The results showed that the differences between femininity and FNE (*z* = −2.22, *p* = 0.013), femininity and social risk (*z* = 2.51, *p* = 0.006), and FNE and social risk-taking (*z* = 1.73, *p* = 0.042) were statistically significant. Therefore, these findings support Hypotheses 2a, b, and 3, in that women who self-reported greater feminine traits experienced more FNE and make fewer risky decisions in the social domain.

### Indirect Effects of Sex on Social Risk-Taking Based on Femininity and Fear of Negative Evaluation

To test whether femininity and FNE mediated the association between sex and social risk-taking (see model 1, Figure 1), we followed the recommendations of Hayes (2017) for testing indirect effects with serial mediators. It is necessary to consider that a significant total effect is not required to obtain a significant indirect effect (Hayes, 2009). According to Hayes (2017), an indirect effect can be interpreted as statistically significant if zero falls outside of a confidence interval. To check our prediction, we used model 6 of the PROCESS macro for SPSS version 3.4.1, with 10.000 bias-corrected bootstrap samples and 95% confidence intervals. We entered sex (0 = male, 1 = female) as the predictor (X), femininity (M1) and FNE (M2) as the mediating variables, and risk-taking in the social domain as the criterion variable (Y). The results showed that the indirect effect was significant, given that the 95% confidence interval around the indirect effect did not contain zero [*B* = −0.010, *SE* = 0.006, 95% CI (−0.023, −0.002)], supporting Hypothesis 4. That is to say, sex (0 = male, 1 = female) was indirectly linked to risk-taking in the social domain, through femininity and FNE. In particular, women (vs. men) self-reported greater feminine traits, which were associated with higher FNE, which in turn was related to making less risky decisions in the social domain (see Figure 1).

It is worthwhile to point out that the pathways through each of the mediators notably were not significant, given that the 95% confidence interval around the indirect effect contained zero in both cases: (a) the indirect effect of sex on social risk-taking through femininity [*B* = −0.001, *SE* = 0.017, 95% CI (−0.033, 0.035)] and (b) the indirect effect of sex on social risk-taking through FNE [*B* = 0.005, *SE* = 0.023, 95% CI (−0.042, 0.049)]. Therefore, femininity and FNE are essential for these pathways to unfold, and the association between them is relevant in this process. Furthermore, as can be observed in Table 3, it should be noted that both mediators accounted for 10% of the variance in the inclination to social risk-taking, instead of 3% or 1% if they were considered independently.

## Discussion

In the present research, we aimed to analyze what variables affect women making risky decisions in the social domain. The findings provide an explication from a gender-based perspective of why there are sex differences in social risk-taking, a controversial question that should be analyzed from this perspective (Zhang et al., 2019). The results show that gender roles (femininity) and FNE – psychosocial variables – are plausible explanatory factors in the relation between women and higher risk-taking in the social domain. Although the majority of research conducted on gender roles and the FNE phenomenon has focused on the sports domain (for a review, see Chalabaev et al., 2013), our work extends a growing body of literature considering risky decision-making as another stereotypically masculine domain (e.g., Cerrato and Cifre, 2018; Morgenroth et al., 2018) in which these variables could determine women’s behavior.

Our findings revealed that women compared to men self-reported greater feminine traits (Hypothesis 1a). This disparity is consistent with social role theory (Eagly, 1987) as well as other studies (e.g., Mueller and Dato-on, 2013), showing that in spite of an increase of women and men in nonstereotypical domains, gender inequality remains in societies (Haines et al., 2016). Indeed, women still consider themselves as primarily responsible for housework and childcare, spending more time on these tasks compared to men, who consider primarily responsible for decision-making tasks (Cerrato and Cifre, 2018). One of the main reasons women continue conforming to their gender roles (femininity) in their behavior is social sanctions that they could receive (Rudman et al., 2012; Eagly and Wood, 2016). Women evaluate themselves positively to the extent that they conform to gender roles or negatively to the extent that they deviate from them, because if they show nonstereotypical behavior, they might experience social sanctions. Indeed, empirical evidence has shown that femininity affects social behavior, increasing avoidance or social insecurity (Cella et al., 2013). In this sense, our results align with previous studies, as femininity was positively associated with FNE (Hypothesis 2a). According to our findings, women who self-reported more feminine traits had more FNE, which could owe to the level of pressure women feel to conform to their gender roles (Dinella et al., 2014) as well as concern about not achieving social standards of femininity (Leary, 1992). For example, women who do not fulfill the role of a mother can experience fear of being evaluated by others as a “bad mother or bad woman” (Liss et al., 2013). Women are constantly evaluated by society, given that they should not disregard their traditional role (i.e., the private sphere) to maintain gender inequality situations.

By contrast, concerning FNE, the results did not show statistically significant differences based on sex, which does not support Hypothesis 2b.This result is not consistent with empirical evidence, whereby women have reported experiencing more FNE (e.g., Biolcati, 2017). Nevertheless, it should be noted that although there were no significant differences, our averages notably showed that women reported more FNE. This pattern of results could be explained by social desirability bias, which can lead women to want to appear good to others (Paulhus, 1984). Currently, women could want to be perceived as feminists given an expansion of the feminist movement in Spain, which has been encouraging women to be nontraditional. Feminist women are seen as more competent (masculinity) and less warm (femininity; Meijs et al., 2019), and so women could feel social pressure to appear more masculine and not show FNE to others.

Concerning social risk-taking, empirical evidence has found differences between the sexes: Women in other studies have made more risky decisions in this domain than men (Blais and Weber, 2006; Lozano et al., 2017; Morgenroth et al., 2018), which our study also found (Hypothesis 1c). Studies have argued that these differences should be interpreted with caution (Zhang et al., 2019), and according to gender stereotypes, given that risk-taking is traditionally associated with stereotypically masculine activity (Morgenroth et al., 2018). Nevertheless, to our knowledge, there are no studies that have tried to explain these differences through a gender-based perspective. Our findings indicate that in women, femininity and FNE are negatively associated with social risk-taking (Hypotheses 2b and 3). Despite the scarce existing literature that associates femininity or FNE with social risk-taking, these findings could be mainly explained by social role theory (Eagly, 1987) and backlash effect (Rudman et al., 2012). Traditionally, women are conditioned to be more cautious and men to be riskier (Carver et al., 2013); thus, if women are involved in a stereotypically masculine domain (risk-taking), they could be concerned about not achieving social standards of femininity (Leary, 1992). Specifically, nonstereotypical women are perceived more negatively than stereotypical men or women (Sutherland et al., 2015) and are more likely to receive social sanctions (Rudman et al., 2012). These differences could also be explained by information processing (e.g., Byrne and Worthy, 2016; Meyers-Levy, 1989): Men process information selectively to make decisions, using specific information that benefits their decisions, whereas women use integrated information processing, taking into account all information in an environment (i.e., social sanctions), even when information can lead them to make bad decisions. The impact of sex on information processing maintains some parallelism with the effect of power (structural variable) on strategies people adopt to achieve their objectives (Schmid et al., 2015). Powerful people – usually men – focus their attention on achieving their goals, regulating their behavior toward them (e.g., Guinote, 2017). By contrast, powerless people – usually women – have a constant need for control, directing their attention to different sources of information (Keltner et al., 2003). Everything being taken into account, men’s behavior could be said to depend only on them, whereas women need the approval of others to carry out their behavior – even more so if their behavior is nonstereotypical. In line with this reasoning, given that women use all information in a context, they could consider the possibility of receiving social sanctions if they do not conform to traditional gender roles and could consequently limit their behavior to their traditional role. In this sense, FNE could be a variable that reflects the fear of social sanctions in feminine women and therefore leads them to make less risky decisions.

Extending prior research that showed that gender stereotypes and FNE can explain women’s behaviors in stereotypically masculine domains (e.g., Chalabaev et al., 2013), such as risk-taking (Morgenroth et al., 2018), we found that women in general make greater risk decisions in the social domain than men do (Hypothesis 1c), in line with previous studies (Blais and Weber, 2006; Lozano et al., 2017; Morgenroth et al., 2018). To explain these sex differences from a gender-based perspective, we tested an integrated serial mediation model that considers both femininity and FNE as explicative variables of social risk-taking. The main findings demonstrated that the association between sex and social risk-taking is mediated by femininity and FNE (Hypothesis 4). That is, women (vs. men) self-reported greater feminine traits, which were associated with higher FNE, which in turn was related to making less risky decisions in the social domain. These results expand the literature on sex differences in social risk-taking by demonstrating a gender confirmation bias in women’s answers. Although women want to make risky decisions in the social domain, such as “moving to a city far away from your extended family,” they fear being judged by others for deviating from their traditional role (femininity). Therefore, until gender roles (femininity) weaken, beliefs about what women should do will not disappear, and neither, therefore, will the negative sanctions women receive if they deviate from those roles. Hence, this work expands evidence on risk-taking in women through social role theory (Eagly, 1987), confirming that gender roles can limit women to stereotypically feminine activities (i.e., in the private sphere). In sum, not all women make more risky decisions in the social domain but those who do not have gender roles more internalized.

### Limitations and Directions for Future Research

Even though the present work contributes to a better understanding of risk-taking by women in the social domain – measured through the DOSPERT scale – it has some limitations that need to be reported. Despite our sample being large, it cannot be regarded as representative of all women, given that the participants were undergraduates. To improve the generalizability of the research results, researchers will need to complete studies based on the general population. Furthermore, participants were not asked to provide their sexual orientation. We recommend future researchers to consider sexual orientation as a control variable, given that previous research has related it with femininity (e.g., Salvati et al., 2019). It would be interesting for future studies to analyze how women self-report feminine traits as a function of sexual orientation and the relationship between those traits and behavior in stereotypically masculine domains. Lastly, the amount of unexplained variance in social risk-taking may suggest that it depends on other variables as well. We recommend future researchers consider including other gender variables, such as sexism attitudes, that are associated with highly traditional roles (e.g., Becker and Wagner, 2009) and could decrease the likelihood to engage in social risk-taking. Likewise, feminist identity could be another explanatory variable for social risk-taking in women. Indeed, feminist women are seen as more competent (masculine) and less warm (feminine; Meijs et al., 2019), which could be associated with less FNE and more risky decisions in the social domain. Feminist women want to confront traditional roles (Weis et al., 2018) and so should not experience fear of social sanctions. Thus, from a gender-based perspective, feminist identity could be a valuable topic in future research on social risk-taking.

### Conclusion

Empirical evidence and theories have demonstrated that cultural stereotypes influence gender-typed behavior. The present work contributes to improvement knowledge of the stereotypes and risk-taking fields, demonstrating that gender roles could explain sex differences in risk-taking, a stereotypically masculine domain. The results confirm that women make more risky decisions in the social domain than men, but they also add a plausible explanation for this sex-based relation. This study provides evidence that women (vs. men) identify themselves as more feminine, which is associated with higher FNE and in turn with making less risky decisions in the social domain. Thus, it seems that those women who have gender roles more internalized make less risky decisions in the social domain. Findings underscore the importance of femininity and FNE to social risk-taking among women. These psychological variables lead to maintaining gender inequality in society – as can be observed in our findings – which decreases the likelihood of women behaving in stereotypically masculine domains.

Furthermore, we agree with previous studies, which indicated that DOSPERT’s sex differences should be interpreted with caution because they could be biased due to gender stereotypes. In this sense, through a gender-based perspective, we have added a plausible explication of these differences through femininity and FNE.

## Data Availability Statement

The raw data supporting the conclusions of this article will be made available by the authors on request to the corresponding author, without undue reservation.

## Ethics Statement

The study is part of a broad project approved by the Ethics Committee of the University of Granada. All procedures performed in study were in accordance with the ethical standards of the University of Granada and with the 1964 Helsinki declaration. Participants provided informed consent to participate in this study.

## Author Contributions

LV-M and FE designed the study and drafted the content of the paper. LV-M collected the data, analyzed and interpreted them. Both the authors contributed to manuscript revision, read, and approved the submitted version.

### Conflict of Interest

The authors declare that the research was conducted in the absence of any commercial or financial relationships that could be construed as a potential conflict of interest.
